# Efficacy and safety of intravenous tenecteplase compared to alteplase before mechanical thrombectomy in acute ischemic stroke: a meta-analysis

**DOI:** 10.1007/s00415-024-12445-7

**Published:** 2024-05-23

**Authors:** Nihong Wu, Thorsten R. Doeppner, Dirk M. Hermann, Janine Gronewold

**Affiliations:** 1 Department of Neurology, University Hospital Essen, Hufelandstraße 55, 45122 Essen, Germany; 2 Department of Neurology, Justus Liebig University Gießen, Gießen, Germany

**Keywords:** Early recanalization, Fibrinolysis, Recanalization therapy, Recombinant tissue-plasminogen activator, Thrombolysis

## Abstract

**Background:**

The benefits and risks of tenecteplase (TNK) versus alteplase (ALT) have recently been assessed in acute ischemic stroke (AIS) patients undergoing mechanical thrombectomy (MT) with diverse results. Due to its high fibrin specificity and lack of excitotoxicity, TNK may have a higher efficacy and safety profile. This study aimed to evaluate the benefits and risks of TNK compared to ALT in AIS patients prior to thrombectomy.

**Methods:**

We systematically searched four key databases, PubMed, Embase, Web of Science and Cochrane Library until January 27, 2024 for clinical studies evaluating the effects of TNK versus ALT in patients with large vessel occlusion undergoing MT. A random-effect meta-analysis was performed following the Preferred Reporting Items for Systematic Reviews and Meta-Analyses (PRISMA) guidelines.

**Results:**

Ten studies involving 3722 patients receiving TNK (1266 patients) or ALT (2456 patients) were included (age: 69.05 ± 14.95 years; 55.64% male). Compared to ALT-treated patients, TNK-treated patients demonstrated significantly higher rates of early recanalization (odds ratio 2.02, 95%-confidence interval 1.20–3.38, p = 0.008) without increased risk of symptomatic intracerebral hemorrhage (1.06, 0.64–1.76, p = 0.82) or intracerebral hemorrhage (1.21, 0.66–2.25, p = 0.54). TNK-treated patients showed similar rates of functional independence at 90 days (1.13, 0.87–1.46, p = 0.37) as ALT-treated patients, but lower rates of mortality within 90 days (0.65, 0.44–0.96, p = 0.03).

**Conclusion:**

TNK is superior to ALT in achieving early recanalization and is associated with lower mortality within 90 days in AIS patients undergoing MT. Compared with ALT, TNK does not significantly alter functional independence at 90 days, symptomatic intracerebral hemorrhage or intracerebral hemorrhage.

**Supplementary Information:**

The online version contains supplementary material available at 10.1007/s00415-024-12445-7.

## Background

Mechanical thrombectomy (MT) has emerged as the treatment of choice for acute ischemic stroke (AIS) with large vessel occlusion, with its effectiveness confirmed in multiple randomized trials [1]. Prior to MT, intravenous thrombolysis is widely recommended. Although some discussion regarding bridging thrombolysis still remains [2–4], current evidence increasingly advocates for thrombolytic therapy in MT-eligible patients [5–9]. Alteplase (also: recombinant tissue-plasminogen activator; ALT) is currently the most commonly used and guideline-recommended thrombolytic agent for patients with AIS within 4.5 h after stroke onset [10]. Although the beneficial effects of ALT on recanalization are certain, ALT may induce toxicity and aggravate ischemic brain injury [11, 12], possibly via secondary hemodynamic disturbances [13, 14].

Tenecteplase (TNK) is a promising thrombolytic agent due to its greater fibrin specificity and longer half-life compared with ALT [15, 16]. Although TNK has not been classified as a recommended class I thrombolytic agent in current stroke guidelines [10], increasing numbers of randomized controlled trials (RCTs) confirm the non-inferiority of TNK to ALT [17–20]. In recent years, studies in AIS started to more systematically compare the efficacy and safety of the two thrombolytic agents [19]. The Australian Stroke Guidelines and the New Zealand Stroke Network recognized TNK, specifically at a dosage of 0.25 mg/kg (maximum 25 mg), as an alternative for ALT in 2019 [21]. By comparing the outcomes of all adult patients treated with ALT in the New Zealand Central Region from January 1 2018 to March 1 2020 with those treated with TNK from March 2 2020 to February 14 2021, it was found that the functional independence at 90 days after switching from ALT to TNK was improved and there was no increase in symptomatic intracranial hemorrhage (ICH, sICH) [21]. In a recent large RCT trial, TNK was non-inferior to ALT in patients with AIS who were eligible for standard intravenous thrombolysis but ineligible for or refused MT [18].

Despite much evidence supporting the use of TNK for thrombolysis in ischemic stroke, there has been limited data on the efficacy and safety of TNK vs. ALT in AIS patients undergoing MT [18, 21, 22]. In a multicenter RCT, the administration of TNK prior to MT was associated with better early recanalization and functional outcome compared with ALT in AIS patients who were treated within 4.5 h of symptom onset [19]. ICH, sICH and mortality in the latter study did not differ between groups. Some studies made similar observations [23–26], whereas other studies could not confirm these findings [16, 27–30]. To provide a comprehensive analysis and to deepen the understanding of the benefits and risks of TNK vs. ALT, we performed a meta-analysis comparing the efficacy and safety of both thrombolytics as bridging agents for MT.

## Methods

### Search strategy

This meta-analysis follows the Preferred Reporting Items for Systematic Reviews and Meta-Analyses (PRISMA) guidelines (see Supplementary File 1). We systematically searched four key databases, PubMed, Embase, Web of Science and the Cochrane Library up to January 27 2024. We conducted a comprehensive search of titles and abstracts using a precise combination of keywords and MeSH terms. These included “Alteplase”, “Tenecteplase”, “Ischemic Stroke”, “Ischaemic Stroke”, “Cerebral Infarction”, “Middle Cerebral Artery Infarction”, “Middle Cerebral Artery Stroke” and “Thrombectomy”, by using the “AND” and “OR” logical operators in conjunction with each other (see Supplementary File 2).

### Inclusion criteria and exclusion criteria

The inclusion criteria were: (1) RCT or cohort study or observational study, (2) conducted in AIS patients over 18 years of age who underwent MT, (3) comparison groups include TNK and ALT groups, (4) study includes indicators of efficacy or safety, (5) study published as full-text manuscript or conference abstract in English, and (6) sample size > 5 per group. Exclusion criteria were: Review, case series, meta-analysis, protocol, letter, editorial, basic research in cells or animals, duplicate database sources, lack of specific numbers for any indicator observed.

### Data extraction

Two experienced researchers (NW and TRD) independently screened the articles and extracted data of the eligible studies, including publication types, study design, country and occluded site, total number of patients, age, sex, thrombolytic dose, outcomes, covariates and main results. Any discrepancies were resolved by discussion among all authors. Primary outcomes were early successful recanalization, defined as successful vascular reperfusion (more than 50% restoration of blood flow in the involved territory) observed at the time of the initial angiographic assessment (modified Thrombolysis in Cerebral Infarction (mTICI) or expanded Thrombolysis in Cerebral Infarction (eTICI) score 2b-3), and safety indicators included sICH and ICH. ICH was defined as any type of ICH assessed by imaging or hemorrhage-related rating scale, sICH was defined as ICH that was temporally related to and directly contributing to the deterioration of the neurological condition [30]. Secondary outcomes were functional independence at 90 days defined as mRS score of 0–2 and all-cause mortality within 90 days.

### Quality assessment

Considering that most of the included studies were non-randomized, we used the Newcastle–Ottawa scale (NOS) for cohort studies to assess the quality of the included studies. Two investigators (NW and TRD) independently assessed the included studies in three ways: cohort selection, cohort comparability, and assessment of outcomes [31]. Any discrepancies were resolved by discussion among all authors. A study with 3 or fewer, 4 to 6, or 7 to 9 stars can be considered low quality, moderate quality, or high quality respectively. Additionally, funnel plots were used to detect potential publication bias and asymmetry was considered present if p < 0.10 (Egger’s test) [32].

### Statistical analysis

In this meta-analysis, outcome data were analyzed using random-effects models. We calculated odds ratios (OR) with corresponding 95% confidence intervals (CI) as measures of effect size. Inter-study heterogeneity was assessed via the I^2^ statistic with thresholds set at ≤ 25% (possible/unclear), > 25% to ≤ 50% (low), > 50% to ≤ 75% (moderate), and > 75% (high) heterogeneity. To address the presence of heterogeneity in treatment outcomes, we conducted subgroup analyses based on several variables: the nature of the trial (randomized vs. non-randomized), its design (double-blind vs. non-blind), its approach (prospective vs. retrospective), the infarction location (anterior circulation vs. posterior circulation vs. anterior + posterior circulation), NOS study quality (high vs. medium) and review system (peer-review vs. no peer-review). Apart from making comparisons between subgroups, we also performed a pooled analysis of only peer-reviewed articles in order to compare them with the overall pooled results, since peer-reviewed articles are considered to have a more rigorous quality review. Additionally, we performed sensitivity analyses excluding single studies to further explore inter-study heterogeneity. Statistical analyses were performed using Review Manager software (RevMan, version 5.4).

## Results

### Literature retrieval

**Fig. 1:**
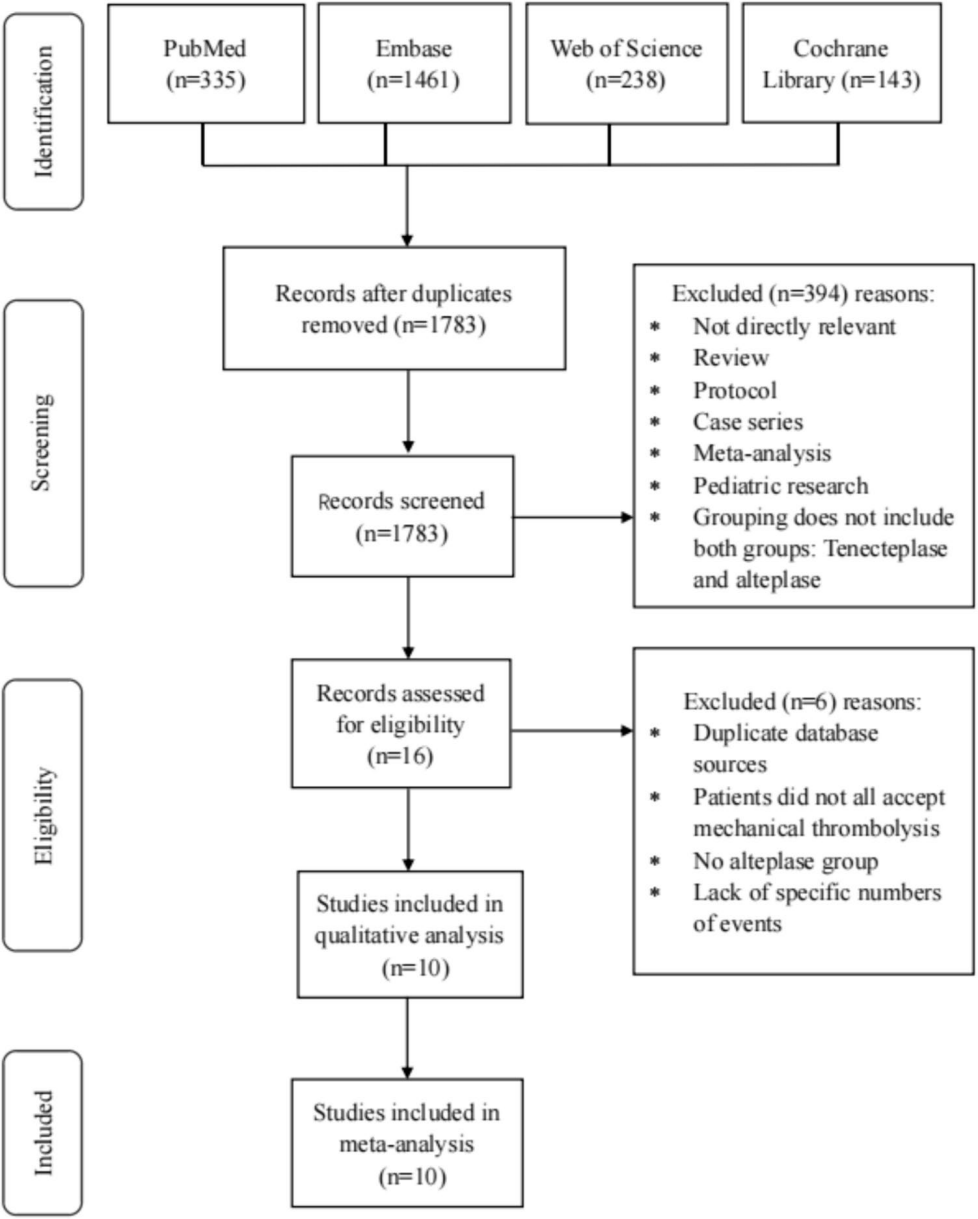
Preferred Reporting Items for Systematic Reviews and Meta-Analyses (PRISMA) flowchart for the included studies. Via thorough analysis of PubMed, Embase, Web of Science and Cochrane library, ten studies were identified which were included in this meta-analysis

We retrieved a total of 2076 publications, including articles and conference abstracts, from the four key databases: PubMed (335), EMBASE (1461), Web of Science (238), and the Cochrane Library (143). Of these, 394 articles were duplicates and the remaining 1783 articles were screened by title, abstract or full text. After reviewing all the screened literature, a total of ten studies were suitable for inclusion in this meta-analysis. The selection process is summarized in Fig. 1, which provides a detailed flowchart of the literature screening methodology.

### Characteristics of included studies

This meta-analysis included ten studies [16, 19, 23–30] with a total of 3722 AIS patients who received MT. Of these, 1266 patients were treated with TNK and 2456 with ALT for thrombolysis. Table 1 shows characteristics of the included studies. Regarding study design, this meta-analysis included two RCTs [19, 30] and eight non-randomized, non-blind cohort studies [16, 23–29]. Eight used a prospective approach [16, 19, 23–25, 27, 28, 30], while two were based on a retrospective approach [26, 29]. Regarding review system, six were peer-reviewed studies [19, 23–25, 28, 30] and four were non-peer-reviewed studies [16, 26, 27, 29]. The largest sample was included in the study by Checkouri et al. (n = 1865) [28], while Marín et al. [29] had the smallest (n = 18). Six studies [16, 19, 23, 26, 27, 29] included both anterior and posterior circulation occlusion, three studies [25, 28, 30] focused on anterior circulation occlusion, and one [24] on posterior circulation occlusion. Thrombolytic doses were highly consistent across studies, using intravenous TNK (0.25 mg/kg) and ALT (0.9 mg/kg). Of note, in one study [24] a broader dose application was introduced in TNK treatment, allowing for 0.25 mg/kg or 0.4 mg/kg.

**Table 1:** Characteristics of included studies

First author (year published)	Publication type, design, country and occluded site	Total patient number	Age, in years (mean ± SD or median (IQR))	Sex, n	Drug dose	Outcome measure	Covariates adjusted for	Main results TNK vs. ALT group
Campbell 2018	Article; Prospective; Australia and New Zealand; ICA, MCA, BA	202	71.15 ± 14.44	Male: 110 Female: 92	TNK, 0.25 mg/kg; ALT, 0.9 mg/kg	ER, sICH, ICH, FI at 90 days, mortality within 90 days; medical records and telephone conversation	NIHSS score and age at baseline	ER: AOR = 2.6; 95% CI, 1.1–5.9; p = 0.02 sICH: OR, 1.0; 95% CI, 0.1–16.2; p = 0.99 ICH: OR, 1.2; 95% CI, 0.4–4.1; p = 0.7 FI at 90 days: AOR, 1.8; 95% CI, 1.0–3.4; p = 0.06 Mortality within 90 days: AOR, 0.4; 95% CI, 0.2–1.1; p = 0.08
Checkouri 2023	Article; Prospective; France; ICA, MCA	1865	70.2 ± 15.27	Male: 917 Female: 948	TNK, 0.25 mg/kg; ALT, 0.9 mg/kg	ER; register based	Age, NIHSS score on admission, time period (before or after 2016), onset-to-IVT time, occlusion site, type of transfer, diabetes mellitus, and type of imaging for ER evaluation, susceptibility vessel sign, and thrombus length	ER: OR, 1.09; 95% CI, 0.83–1.44; p = 0.52
Hendrix 2023	Article; Prospective; American; ICA, MCA, BA	148	73.31 ± 14.39	Male: 76 Female: 72	TNK, 0.25 mg/kg; ALT, 0.9 mg/kg	ER, sICH, ICH, FI at 90 days, mortality within 90 days; medical records	Baseline NIHSS score, CT-Alberta Stroke Program Early CT Score (CT-ASPECTS), primary presentation to CSC, and arterial hypertension	ER: 23.5% vs. 10.3%; p = 0.032; sICH: 3.9% vs. 1.0%; p = 0.273 ICH: 5.9% vs. 7.2%; p = 1.000 FI at 90 days: 59.6% vs. 61.1%; p = 0.865 Mortality within 90 days: 19.1% vs. 21.1%; p = 0.791
Bala 2023	Article; Prospective; Canada; ICA, MCA	93	67.54 ± 13.83	Male: 63 Female: 30	TNK, 0.25 mg/kg; ALT, 0.9 mg/kg	ER, sICH, ICH, FI at 90 days, mortality within 90 days; register based and telephone conversation	Age, baseline NIHSS score, stroke onset-to-arterial access time, and occlusion location at baseline (ICA, M1-MCA, and M2-MCA), participating site, and carotid stenting	ER: 8.2% vs. 19.0%; p = 0.21 sICH: 2.0% vs. 0%; p = 0.99 ICH: 22.0% vs. 32.6%; p = 0.35 FI at 90 days: AOR, 1.53; 95% CI, 0.51–4.55 Mortality within 90 days: AOR, 0.55; 95% CI, 0.13–2.30
Marnat 2023	Article; Prospective; France; Anterior circulation	753	65.13 ± 13.68	Male: 536 Female: 217	TNK, 0.25 mg/kg; ALT, 0.9 mg/kg	ER, sICH, ICH, FI at 90 days, mortality within 90 days; register based	Age, gender, direct admission to CSC, unknow onset time, medical history, admission systolic blood pressure, admission NIHSS score, admission ASPECTS, pre-stroke mRS > 1, occlusion site, stroke etiology, thrombectomy performed, and process times	ER: 13.1% vs. 3.5%; p < 0.001 sICH: 11.5% vs. 10.9%; p = 0.74 ICH: 66.4% vs. 46.9%; p < 0.001 FI at 90 days: 49.4% vs. 47.1%; p = 0.48 Mortality within 90 days: 11.5% vs. 18.1%; p = 0.008
Alemseged 2021	Article; Prospective; Australia, New Zealand, Europe, United States; BA	110	68.73 ± 14.0	Male: 70 Female: 40	TNK, 0.25 or 0.4 mg/kg; ALT, 0.9 mg/kg	ER, sICH, ICH, FI at 90 days; register based	Age, NIHSS, needle-to-arterial-puncture time, and cardioembolic etiology	ER: RR, 4.0; 95% CI, 1.3–12; p = 0.02 sICH: 0% vs. 1%; p = 0.99 ICH: 0% vs. 3%; p = 0.99 FI at 90 days: RR, 1.2; 95% CI, 0.7–2.0; p = 0.5
Marín 2023	Conference abstract; Retrospective; Spain; Anterior + posterior circulation	18	67.31 ± 17.16	Male: 9 Female: 9	TNK, 0.25 mg/kg; ALT, 0.9 mg/kg	sICH, ICH; medical records	None	sICH: OR, 0.24; p = 0.37 ICH: OR, 1.67; p = 0.69
Vetra 2022	Conference abstract; Retrospective; Latvia; ICA, MCA, BA	130	73 (63–80)	Male: 66 Female: 64	TNK, 0.25 mg/kg; ALT, 0.9 mg/kg	ER; medical records	Age, sex, onset-to-EVT-time and baseline NIHSS	ER: 22.2% vs 8.2%
Cruz Culebras 2021	Conference abstract; Prospective; Spain; Anterior + posterior circulation	31	67.85 ± 17.05	Male: 16 Female: 15	TNK, 0.25 mg/kg; ALT, 0.9 mg/kg	ER, sICH, FI at 90 days, mortality within 90 days; medical records and follow-up	NIHSS, age, presence of special situations (wake-up stroke, unknown onset, or known onset > 4.5 h), systolic and diastolic blood pressure before fibrinolysis	ER: 100% vs. 87% sICH: 0% vs. 0% FI at 90 days: 52% vs. 41% Mortality within 90 days: 5.8% vs. 14%
Ainz Gomez 2021	Conference abstract; Prospective; Spain; Anterior + posterior circulation	372	73 (61–80)	Male: 208 Female: 164	TNK, 0.25 mg/kg; ALT, 0.9 mg/kg	ER, sICH; medical records and follow-up	Age, door-to-needle time, occlusion site, ASPECTS, and NIHSS	ER: 18.3% vs. 15.1% sICH: 5% vs. 5.4%

*AOR* adjusted odds ratio, *BA* basilar artery, *CSC* comprehensive stroke centers, *CI* confidence interval, *ER* early recanalization, *EVT* endovascular treatment, *FI* functional independence, *IQR* interquartile range, *ICA* internal carotid artery, *ICH* intracranial hemorrhage, *IVT* intravenous thrombolysis, *MCA* middle cerebral artery, *NIHSS* National Institutes of Health Stroke Scale, *NA* not available, *OR* odds ratio, *RR* risk ratio, *SD* standard deviation, *sICH* symptomatic intracranial hemorrhage, *TNK* tenecteplase, *ALT* alteplase, *vs.* versus

### Quality assessment

Eight studies [16, 19, 23–25, 27, 28, 30] were classified as high quality (NOS scores 7–9), while two studies [26, 29] were medium quality (NOS scores 4–6). Detailed NOS scores for each study are shown in Table 2.

**Table 2:** The Newcastle–Ottawa Scale (NOS) score of included studies

First author (year published)	Selection				Comparability	Outcomes			Total score
	Representativeness of exposed cohort	Selection of nonexposed cohort	Ascertainment of exposure	Outcome not present at the start of the study		Assessment of outcomes	Length of follow-up	Adequacy of follow-up	
Campbell (2018)	*	*	*	*	**	*	*	*	*********
Checkouri (2023)	0	0	*	*	**	*	*	*	*******
Hendrix (2023)	0	0	*	*	**	*	*	*	*******
Bala (2023)	*	*	*	*	**	*	*	*	*********
Marnat (2023)	0	0	*	*	**	*	*	*	*******
Alemseged (2021)	0	0	*	*	**	*	*	*	*******
Marín (2023)	*	*	*	0	0	*	0	0	****
Vetra (2022)	*	*	*	0	**	*	0	0	******
Cruz Culebras (2021)	*	*	*	*	**	*	*	0	********
Ainz Gomez (2021)	*	*	*	*	**	*	*	0	********

### Risk of bias

Funnel plots (Supplementary File 3) suggested no significant publication bias for the outcome of early recanalization, sICH, ICH and functional independence at 90 days and mortality within 90 days (all p > 0.1, Egger’s test).

### Overall analysis of primary outcomes

**Fig. 2:**
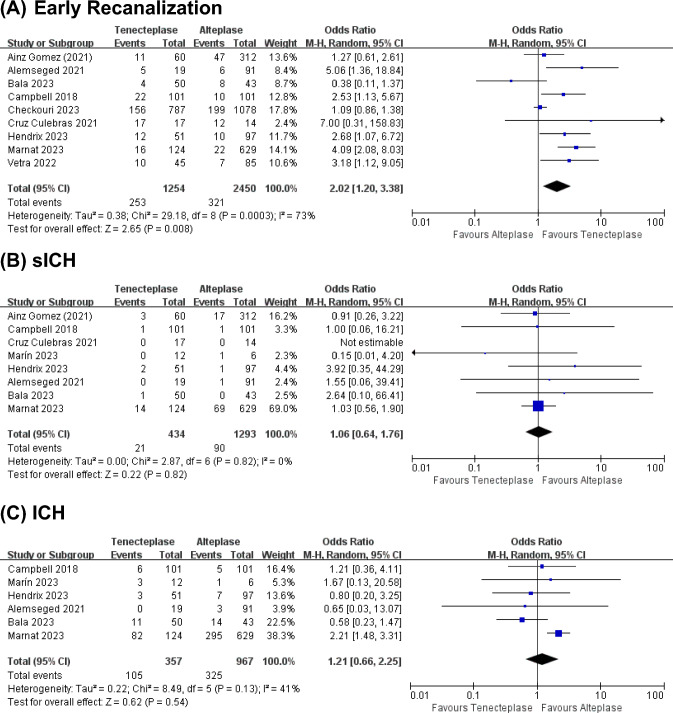
Forest plots for primary outcomes. **A** Early recanalization, **B** symptomatic intracranial hemorrhage (sICH) and **C** intracranial hemorrhage (ICH) in each of the studies and the pooled data analysis. Data are odds ratios (ORs) with 95% confidence intervals (CIs)

Nine studies evaluated early recanalization, eight investigated sICH rates and six evaluated ICH rates, as shown in Fig. 2. TNK treatment achieved a significantly higher early recanalization rate (20.2%) compared to ALT (13.1%) (weighted OR 2.02; 95%-CI 1.20–3.38, p = 0.02, Fig. 2A), with moderate heterogeneity (I^2^ = 73%, Fig. 2A). However, the rate of sICH (TNK + MT 4.8%, ALT + MT 7.0%; 1.06, 0.64–1.76, p = 0.82, Fig. 2B) and ICH (TNK + MT 29.4%, ALT + MT 33.6%; 1.21, 0.66–2.25, p = 0.54, Fig. 2C) was not significantly different, with no and low heterogeneity (I^2^ = 0% and 41%, respectively).

### Overall analysis of secondary outcomes

**Fig. 3:**
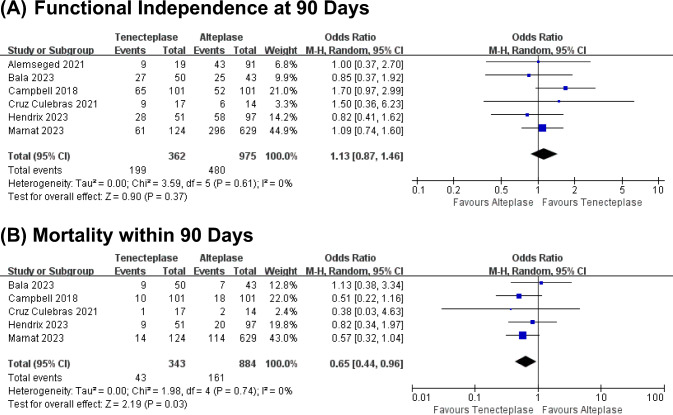
Forest plots for secondary outcomes. **A** Functional independence at 90 days and **B** mortality within 90 days in each study and the pooled data analysis. Data are ORs with 95% CIs

Six studies analyzed functional independence at 90 days and five analyzed mortality within 90 days. TNK treatment exhibited comparable functional independence at 90 days to ALT (1.13, 0.87–1.46, p = 0.37, Fig. 3A), with no heterogeneity (I^2^ = 0%, Fig. 3A). Yet, TNK treatment was associated with lower mortality within 90 days (0.65, 0.44–0.96, p = 0.03, Fig. 3B), with no heterogeneity across the studies (I^2^ = 0%, Fig. 3B).

### Subgroup analyses

**Fig. 4:**
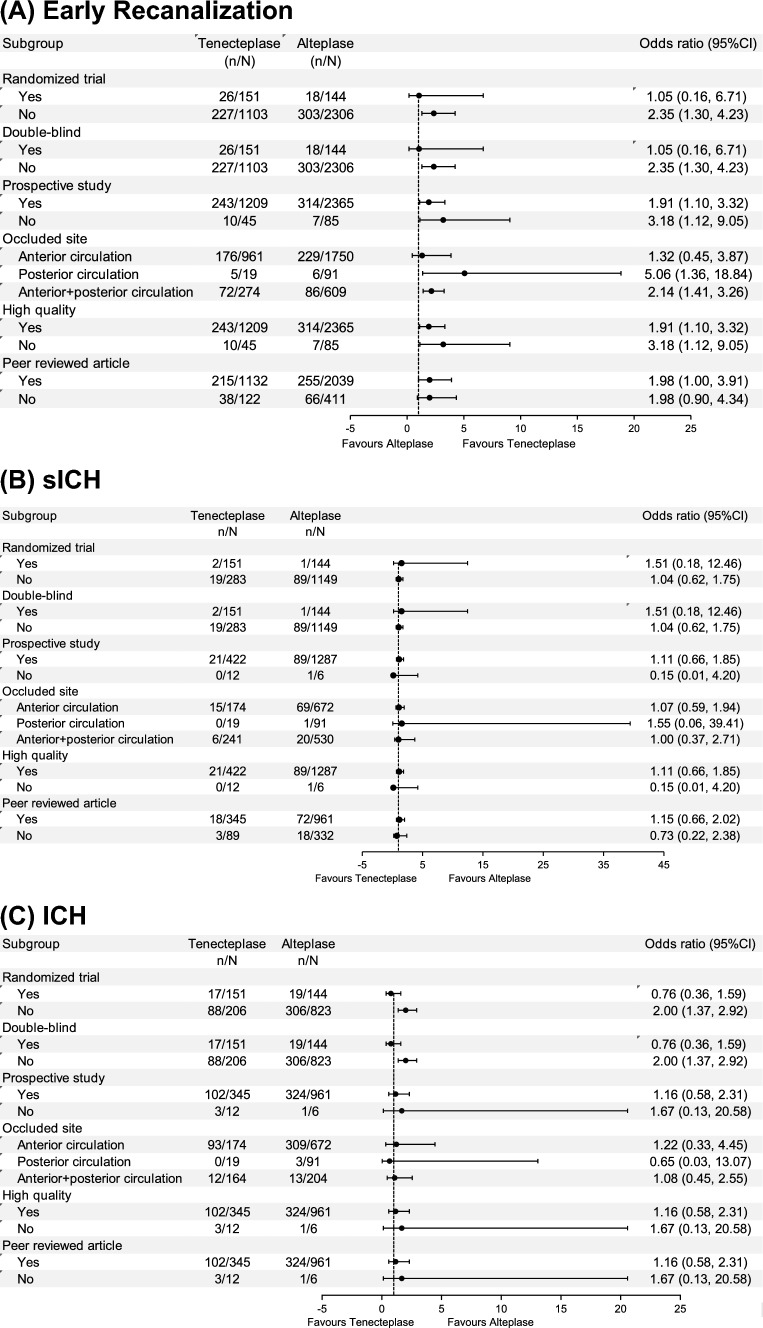
Subgroup analyses for primary outcomes. **A** Early recanalization, **B** sICH and **C** ICH in the pooled data analysis stratified by study characteristics. Data are odds ratios (ORs) with 95% confidence intervals (CIs). n represents the number of events analyzed in the group, while N represents the total patient size of the group

Regarding early recanalization, our findings indicate a uniform treatment effect across all subgroups. The trend of the results consistently favored TNK treatment across all subgroups, although the ORs for TNK treatment were not significant in randomized trials, double-blind studies and patients with occlusion of the intracranial anterior circulation (Fig. 4).

For the outcome of ICH, our analysis did not reveal any treatment effect heterogeneity among the subgroups study approach, occluded site, and NOS study quality, and review system. Notably, the comparison between double-blind randomized trials and non-randomized, non-blind trials showed a significant difference in ICH rates. In double-blind randomized trials, ICH rates did not significantly differ between TNK and ALT treatments, whereas in non-randomized, non-blind trials, the ICH rate was significantly higher for ALT compared to TNK (Fig. 4).

**Fig. 5:**
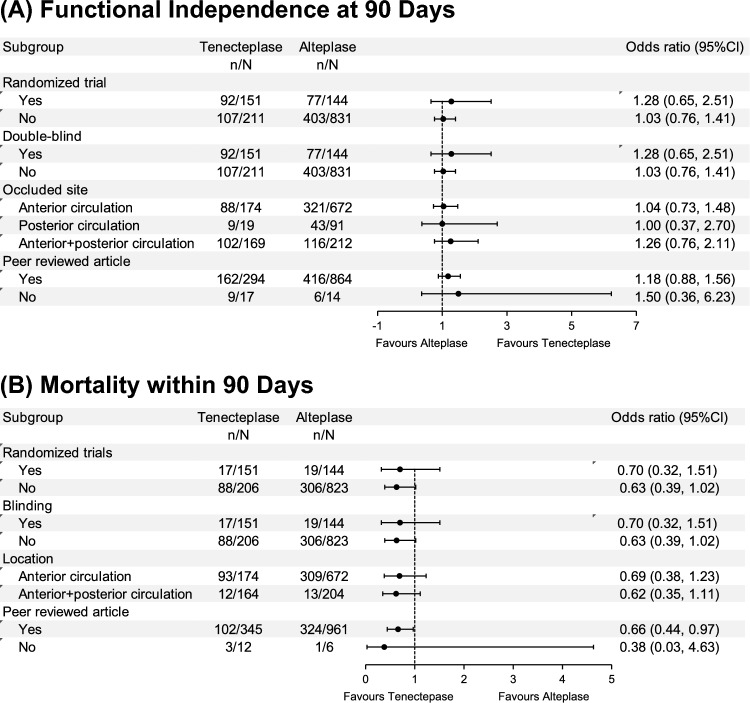
Subgroup analyses for secondary outcomes. **A** Functional independence at 90 days and **B** mortality within 90 days in the pooled data analysis stratified by study characteristics. Data are odds ratios (ORs) with 95% confidence intervals (CIs). n represents the number of events analysed in the group, while N represents the total patient size the group

Regarding mortality within 90 days, our analysis did not reveal any treatment effect heterogeneity across all examined subgroups except for the subgroup concerning publication status. Overall, the TNK group exhibited lower mortality within 90 days, although differences in mortality were not significant among non-peer-reviewed studies (Fig. 5).

For the outcomes of sICH and functional independence at 90 days, our analyses did not reveal any treatment effect heterogeneity across all examined subgroups, as represented in Figs. 4 and 5, respectively.

When we performed a meta-analyses including only data from published peer-reviewed articles (that is excluding conference abstracts) and compared the results with the overall meta-analysis results included all ten studies (peer-reviewed articles plus conference abstracts without peer-review), we obtained similar pooled results for the primary and secondary outcomes, as shown in Supplementary File 4 and Supplementary File 5, with the heterogeneity of results on early recanalization and ICH being slightly increased. TNK treatment was again associated with a higher early recanalization rate compared to ALT treatment (1.98; 1.00–3.91, p = 0.05, Supplementary File 4A) and reduced mortality within 90 days (0.66, 0.44–0.97, p = 0.04, Supplementary File 5B). Compared to ALT, TNK exhibited comparable rates of sICH (1.15, 0.66–2.02, p = 0.62, Supplementary File 4B), ICH (1.16, 0.58–2.31, p = 0.68, Supplementary File 4C) and functional independence at 90 days (1.11, 0.86–1.45, p = 0.42, Supplementary File 5A).

### Sensitivity analyses

Considering inter-study heterogeneity, we subsequently conducted sensitivity analyses by excluding single studies to evaluate the individual impact of each study on the pooled overall effect size of early recanalization and ICH. In these analyses, we found that the pooled effects for early recanalization remained stable following single study removal from analyses.

While pooled analyses showed no statistically significant difference in ICH rates between TNK and ALT treatment, the study by Bala et al. [30] was the main source of heterogeneity affecting the ICH results: Excluding this study significantly reduced heterogeneity from 41 to 0%, and altered the results from no statistically significant difference between the two groups to a significantly higher ICH rate in the ALT group than in the TNK group. This may be due to the fact that the hemorrhage rates in the TNK and ALT groups in the study by Bala et al. were comparable and accounted for 22.5% in the weighted analyses, partially offsetting the difference between the two groups. It is noteworthy that 31.2% of patients in the study by Bala et al. [30] were treated for carotid artery lesions by stenting during thrombolysis bridging to MT, which could also potentially affect the ICH rate between the two groups. The authors recommended that carotid stenting timing needs to be further investigated (see Supplementary File 6).

## Discussion

This meta-analysis investigated the efficacy and safety of TNK vs. ALT in patients with large vessel occlusion undergoing MT, aiming to provide more comprehensive information for clinical decision-making. Our results revealed that intravenous TNK more frequently achieved successful than ALT, which was the primary efficacy outcome, without increasing the risk of sICH and ICH, which were the primary safety outcomes. Regarding the secondary outcomes, TNK was associated with reduced mortality within 90 days and exhibited comparable functional independence at 90 days. The enhanced recanalization potentially explains the reduced mortality, albeit direct impacts of the improved recanalization on sICH, ICH and functional independence were not apparent. To ensure uniformity in the evaluation process, we applied the NOS assessment used for cohort studies [31], which might have led to a degree of underestimation of quality for the retrospective studies [26, 29]. Overall, all the included literature was of medium to high quality.

For the outcome of early recanalization, subgroup analyses showed a high degree of consistency across included studies. Sensitivity analyses which excluded single studies, further affirmed the robustness of the meta-analysis results for the outcome of early recanalization. However, the study by Bala et al. [30] reported an observation different from the other studies with a higher early recanalization rate in the ALT than in the TNK cohort, although this did not reach statistical significance (18.6% vs. 8%, p = 0.21). This deviation may be attributed to the concurrent administration of emergency carotid stenting. The higher fibrin specificity and longer plasma half-life of TNK compared to ALT [11] might provide a better thrombolytic capacity and thus improve early recanalization. Besides, TNK offers the advantage of single-bolus dosing [33], which not only facilitates a more timely and convenient administration but may also accelerate the onset of recanalization compared to ALT. ALT, in contrast, requires a 1–3-h infusion period [33], potentially delaying recanalization. Such differences in administration speed could explain the delay in recanalization rates observed with ALT when compared to TNK, underscoring the practical benefits of TNK in clinical settings.

Evidence from previous meta-analyses in AIS patients consistently suggests that the efficacy and safety of TNK is not inferior to ALT [22, 34–36]. However, given the substantial impact of MT on AIS outcome [2], many studies have intentionally excluded patients undergoing MT from their study cohorts [22, 34–36]. As a result, patients receiving MT were underrepresented in meta-analyses. Nonetheless, bridging thrombolysis is a common treatment in AIS. Therefore, it is clinically important to assess the efficacy and safety of the use of TNK and ALT in MT patients. Our meta-analysis, despite detecting no significant impact on functional independence at 90 days, showed that TNK treatment was associated with a lower mortality within 90 days than ALT treatment. The lower mortality rate observed in the TNK group compared to the ALT group can be attributed primarily to higher recanalization rates with similar risks of hemorrhage. Additional RCTs will need to replicate this finding.

Although the pooled analysis revealed no significant difference in rates of sICH and overall ICH following thrombolysis with TNK and ALT, the subgroup analysis for ICH within four non-randomized trials demonstrated a higher ICH incidence in the ALT than TNK group. Several factors could influence the risk of brain hemorrhage. Firstly, TNK's higher fibrin specificity may reduce hemorrhage risks by limiting circulating plasminogen activation and degradation of fibrinogen [15, 33]. Additionally, the absence of data on antiplatelet use during or after MT could also impact ICH rates. As a potential mechanism, ALT might elevate pro-inflammatory signals in the ischemic brain, thereby contributing to ICH via blood–brain barrier disruption [37]. The occurrence of ICH remains a significant concern in ALT treated AIS patients [38]. As a genetically modified ALT variant, TNK represents a potential alternative [39]. The mechanisms underlying the safety of TNK deserves attention in AIS patients undergoing MT.

Besides TNK, reteplase has been approved by the U.S. Food and Drug Administration (FDA) for the treatment of ST-segment elevation myocardial infarction [40]. Although TNK has been increasingly studied in ischemic stroke, studies of the safety and efficacy of reteplase in ischemic stroke remain limited. Compared with TNK and ALT, the fibrinolytic speed of reteplase is slower, and the risk of ICH may be lower [41]. The results of a recently published phase II RCT showed similar mortality, sICH and functional outcome at 90 days in AIS patients for reteplase and ALT [42], but the sample size was small. Further studies on the benefits and risks of reteplase in larger trials are needed.

## Limitations

This meta-analysis has some limitations: First, to get the most comprehensive information, conference abstracts were included in this meta-analysis because there were limited peer-reviewed studies available. This approach has a major advantage, since it reduces publication bias, which favors studies with beneficial over those with neutral or detrimental findings to be published. However, we also performed a meta-analysis restricted to peer-reviewed articles, which confirmed that TNK was not inferior in efficacy and safety to ALT. Second, while nine of the included studies reported consistent TNK doses (0.25 mg/kg), there is still a possibility of some heterogeneity related to drug dosing, as one study [24] included a higher TNK dose (0.4 mg/kg). Third, when comparing the recanalization rates between the TNK and rt-PA groups, it is noteworthy that although the TNK group still exhibited a higher recanalization rate in RCTs and studies on anterior circulation strokes, it did not reach statistical significance. The absence of significant differences in the RCTs can be attributed to the small number of RCTs, which had limited sample sizes. Only one observational study was specifically performed in anterior circulation strokes. Additional RCTs will have to provide more definitive insights into the comparative effects of TNK vs. rt-PA in AIS patients eligible for MT.

## Conclusions

In AIS patients undergoing MT, TNK demonstrates superior ability over ALT in achieving early recanalization, which is associated with lower mortality within 90 days. Compared with ALT, TNK does not significantly alter functional independence at 90 days, sICH rate, or ICH rate when administered as bridging agent prior to MT.

### Supplementary Information

Below is the link to the electronic supplementary material.

Supplementary file1 (PDF 371 KB)

## Data Availability

The data included into this study are accessible to researchers upon reasonable request.
