# Supplementary material for: Efficacy and safety of intravenous tenecteplase compared to alteplase before mechanical thrombectomy in acute ischemic stroke: a meta-analysis
Source: J Neurol. 2024 May 23;271(7):3928–41. doi: 10.1007/s00415-024-12445-7 (PMC11233346; doi:10.1007/s00415-024-12445-7)
Supplement: Supplementary file 1 — Supplementary file1 (PDF 371 KB) [file 415_2024_12445_MOESM1_ESM.pdf]

Supplementary File 1 PRISMA 2020 Checklist

Section and Topic	Item #	Checklist item	Location where item is reported
**TITLE**			
Title	1	Identify the report as a systematic review.	Page 1
**ABSTRACT**			
Abstract	2	See the PRISMA 2020 for Abstracts checklist.	Page 2
**INTRODUCTION**			
Rationale	3	Describe the rationale for the review in the context of existing knowledge.	Page 3
Objectives	4	Provide an explicit statement of the objective(s) or question(s) the review addresses.	Page 3
**METHODS**			
Eligibility criteria	5	Specify the inclusion and exclusion criteria for the review and how studies were grouped for the syntheses.	Page 4-5
Information sources	6	Specify all databases, registers, websites, organisations, reference lists and other sources searched or consulted to identify studies. Specify the date when each source was last searched or consulted.	Page 4, Supplementary File 2
Search strategy	7	Present the full search strategies for all databases, registers and websites, including any filters and limits used.	Page 4
Selection process	8	Specify the methods used to decide whether a study met the inclusion criteria of the review, including how many reviewers screened each record and each report retrieved, whether they worked independently, and if applicable, details of automation tools used in the process.	Page 4
Data collection process	9	Specify the methods used to collect data from reports, including how many reviewers collected data from each report, whether they worked independently, any processes for obtaining or confirming data from study investigators, and if applicable, details of automation tools used in the process.	Page 4
Data items	10a	List and define all outcomes for which data were sought. Specify whether all results that were compatible with each outcome domain in each study were sought (e.g. for all measures, time points, analyses), and if not, the methods used to decide which results to collect.	Page 4
	10b	List and define all other variables for which data were sought (e.g. participant and intervention characteristics, funding sources). Describe any assumptions made about any missing or unclear information.	Page 4
Study risk of bias assessment	11	Specify the methods used to assess risk of bias in the included studies, including details of the tool(s) used, how many reviewers assessed each study and whether they worked independently, and if applicable, details of automation tools used in the process.	Page 4-5
Effect measures	12	Specify for each outcome the effect measure(s) (e.g. risk ratio, mean difference) used in the synthesis or presentation of results.	Page 5
Synthesis methods	13a	Describe the processes used to decide which studies were eligible for each synthesis (e.g. tabulating the study intervention characteristics and comparing against the planned groups for each synthesis (item #5)).	Page 5
	13b	Describe any methods required to prepare the data for presentation or synthesis, such as handling of missing summary statistics, or data conversions.	Page 5
	13c	Describe any methods used to tabulate or visually display results of individual studies and syntheses.	Page 5
	13d	Describe any methods used to synthesize results and provide a rationale for the choice(s). If meta-analysis was performed, describe the model(s), method(s) to identify the presence and extent of statistical heterogeneity, and software package(s) used.	Page 5
	13e	Describe any methods used to explore possible causes of heterogeneity among study results (e.g. subgroup analysis, meta-regression).	Page 5
	13f	Describe any sensitivity analyses conducted to assess robustness of the synthesized results.	Page 5
Reporting bias assessment	14	Describe any methods used to assess risk of bias due to missing results in a synthesis (arising from reporting biases).	Page 5
Certainty assessment	15	Describe any methods used to assess certainty (or confidence) in the body of evidence for an outcome.	Page 4-5
**RESULTS**			

Section and Topic	Item #	Checklist item	Location where item is reported
Study selection	16a	Describe the results of the search and selection process, from the number of records identified in the search to the number of studies included in the review, ideally using a flow diagram.	Figure 1
	16b	Cite studies that might appear to meet the inclusion criteria, but which were excluded, and explain why they were excluded.	Figure 1
Study characteristics	17	Cite each included study and present its characteristics.	Table 1
Risk of bias in studies	18	Present assessments of risk of bias for each included study.	Page 6
Results of individual studies	19	For all outcomes, present, for each study: (a) summary statistics for each group (where appropriate) and (b) an effect estimate and its precision (e.g. confidence/credible interval), ideally using structured tables or plots.	Figure 2-3, Supplementary File 4-5
Results of syntheses	20a	For each synthesis, briefly summarise the characteristics and risk of bias among contributing studies.	Table 1, Supplementary File 3
	20b	Present results of all statistical syntheses conducted. If meta-analysis was done, present for each the summary estimate and its precision (e.g. confidence/credible interval) and measures of statistical heterogeneity. If comparing groups, describe the direction of the effect.	Page 5-8, Figure 2-5, Supplementary File 4-5
	20c	Present results of all investigations of possible causes of heterogeneity among study results.	Page 6-8, Figure 4-5
	20d	Present results of all sensitivity analyses conducted to assess the robustness of the synthesized results.	Page 6-8, Supplementary File 6
Reporting biases	21	Present assessments of risk of bias due to missing results (arising from reporting biases) for each synthesis assessed.	Page 5-8
Certainty of evidence	22	Present assessments of certainty (or confidence) in the body of evidence for each outcome assessed.	Table 2
**DISCUSSION**			
Discussion	23a	Provide a general interpretation of the results in the context of other evidence.	Page 8-10
	23b	Discuss any limitations of the evidence included in the review.	Page 9-10
	23c	Discuss any limitations of the review processes used.	Page 9-10
	23d	Discuss implications of the results for practice, policy, and future research.	Page 9
**OTHER INFORMATION**			
Registration and protocol	24a	Provide registration information for the review, including register name and registration number, or state that the review was not registered.	The review was not registered
	24b	Indicate where the review protocol can be accessed, or state that a protocol was not prepared.	A protocol was not prepared
	24c	Describe and explain any amendments to information provided at registration or in the protocol.	None
Support	25	Describe sources of financial or non-financial support for the review, and the role of the funders or sponsors in the review.	Page 10
Competing interests	26	Declare any competing interests of review authors.	Page 10
Availability of data, code and other materials	27	Report which of the following are publicly available and where they can be found: template data collection forms; data extracted from included studies; data used for all analyses; analytic code; any other materials used in the review.	Supplementary File

From: Page MJ, McKenzie JE, Bossuyt PM, Boutron I, Hoffmann TC, Mulrow CD, et al. The PRISMA 2020 statement: an updated guideline for reporting systematic reviews. BMJ 2021;372:n71. doi: 10.1136/bmj.n71

For more information, visit: http://www.prisma-statement.org/

Supplementary File 2 Search Strategy

Database: PubMed (335); Searched online: January 27 2024
Search Strategy:
(((Alteplase [Title/Abstract]) OR (Tenecteplase [Title/Abstract])) AND (((((Ischemic Stroke [Title/Abstract]) OR (Ischaemic Stroke [Title/Abstract])) OR (Cerebral Infarction [Title/Abstract])) OR (Middle Cerebral Artery Infarction [Title/Abstract])) OR (Middle Cerebral Artery Stroke [Title/Abstract]))) AND (Thrombectomy [Title/Abstract])

Database: EMBASE (1461); Searched online: January 27 2024
Search Strategy:
#1 'alteplase'/exp (23791)
#2 'tenecteplase':ab,ti (1387)
#3 'ischemic stroke':ab,ti (109966)
#4 'ischaemic stroke':ab,ti (14898)
#5 'cerebral infarction':ab,ti (25512)
#6 'middle cerebral artery infarction':ab,ti (863)
#7 'middle cerebral artery stroke':ab,ti (669)
#8 'thrombectomy':ab,ti (28712)
#9 #1 OR #2 (24588)
#10 #3 OR #4 OR #5 OR #6 OR #7 (145754)
#11 #8 AND #9 AND #10 (1461)

Database: Web of Science (238); Searched online: January 27 2024
Search Strategy:
AB=(('alteplase' OR ' tenecteplase')) AND AB=('ischemic stroke' OR 'ischaemic stroke' OR 'cerebral infarction' OR 'middle cerebral artery infarction' OR ' middle cerebral artery stroke ') AND AB='thrombectomy'

Database: Cochrane Library (143); Searched online: January 27 2024
Search Strategy:
#1 MeSH descriptor: [Tissue Plasminogen Activator] explode all trees (2180)
#2 ('Tenecteplase'):ti,ab,kw (524)
#3 MeSH descriptor: [Ischemic Stroke] explode all trees (1063)
#4 ('Ischaemic stroke'):ti,ab,kw OR ('Cerebral infarction'):ti,ab,kw OR ('Middle cerebral artery infarction'):ti,ab,kw OR ('Middle cerebral artery stroke'):ti,ab,kw (23710)
#5 MeSH descriptor: [Thrombectomy] explode all trees (1063)
#6 #1 OR #2 (2493)
#7 #3 OR #4 (23740)
#8 #5 AND #6 AND #7 (143)

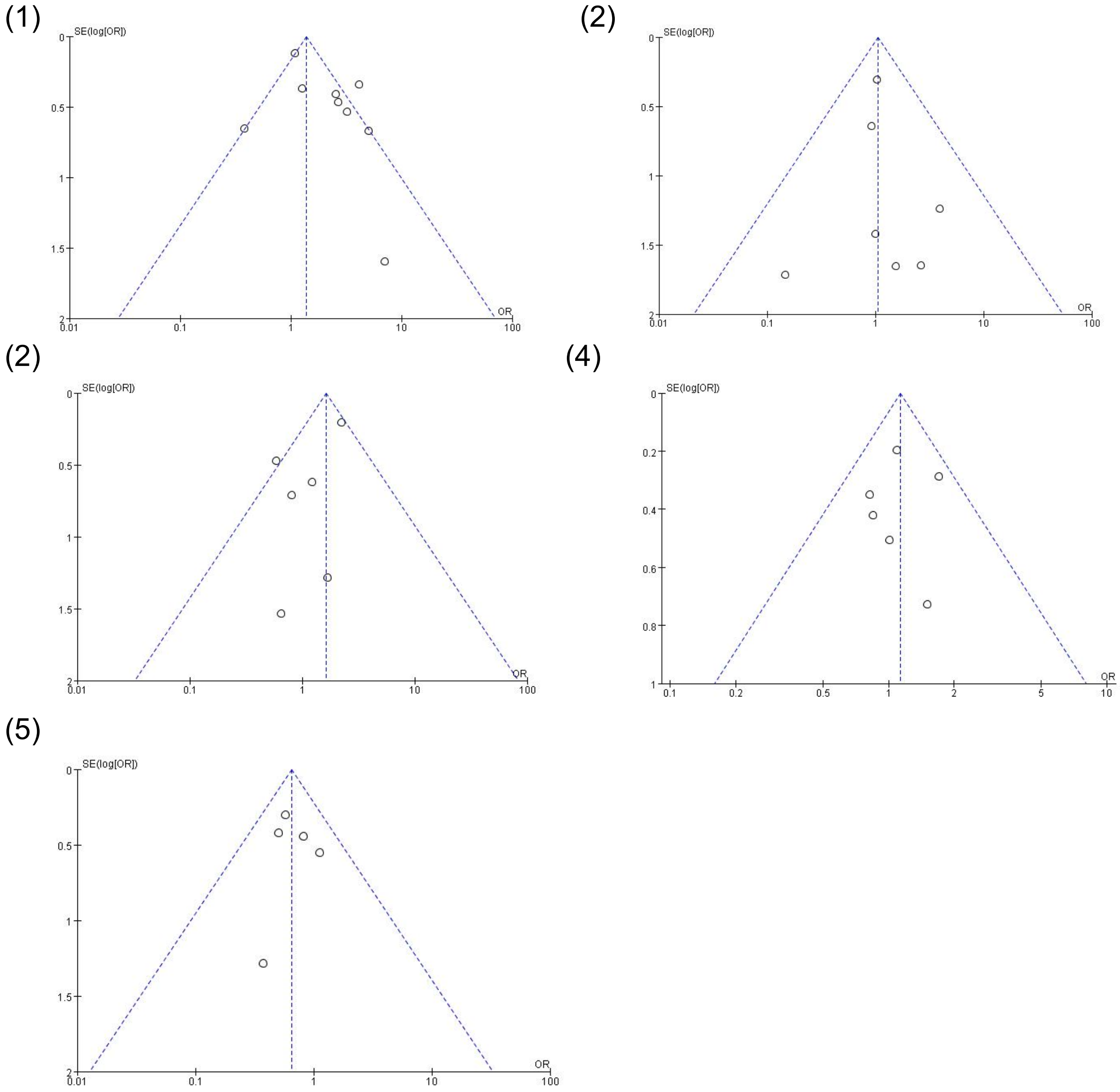

Supplementary File 3 Funnel Plot for (1) Early Recanalization (2) sICH (3) ICH (4) Functional Independence at 90 Days (5) Mortality within 90 Days

(1) Early recanalization: Egger's test revealed an intercept of 1.204 (p = 0.352), suggesting no significant evidence of publication bias.
(2) sICH: Egger's test revealed an intercept of 0.071 (p = 0.953), suggesting no significant evidence of publication bias.
(3) ICH: Egger's test revealed an intercept of 0.286 (p = 0.577), suggesting no significant evidence of publication bias.
(4) Functional independence at 90 days: Egger's test revealed an intercept of -0.0139 (p = 0.9705), suggesting no significant evidence of publication bias.
(5) Mortality within 90 days: Egger's test revealed an intercept of -0.117 (p = 0.774), suggesting no significant evidence of publication bias.
sICH: Symptomatic intracranial hemorrhage, ICH: Intracranial hemorrhage

(1) Early Recanalization

Study or Subgroup	Tenecteplase Events	Tenecteplase Total	Alteplase Events	Alteplase Total	Weight	Odds Ratio M-H, Random, 95% CI
Alemseged 2021	5	19	6	91	12.4%	5.06 [1.36, 18.84]
Bala 2023	4	50	8	43	12.7%	0.38 [0.11, 1.37]
Campbell 2018	22	101	10	101	17.4%	2.53 [1.13, 5.67]
Checkouri 2023	156	787	199	1078	22.5%	1.09 [0.86, 1.38]
Hendrix 2023	12	51	10	97	16.2%	2.68 [1.07, 6.72]
Marnat 2023	16	124	22	629	18.8%	4.09 [2.08, 8.03]
**Total (95% CI)**		**1132**		**2039**	**100.0%**	**1.98 [1.00, 3.91]**
Total events	215		255			

Heterogeneity: Tau² = 0.52; Chi² = 25.68, df = 5 (P = 0.0001); I² = 81%

Test for overall effect: Z = 1.97 (P = 0.05)

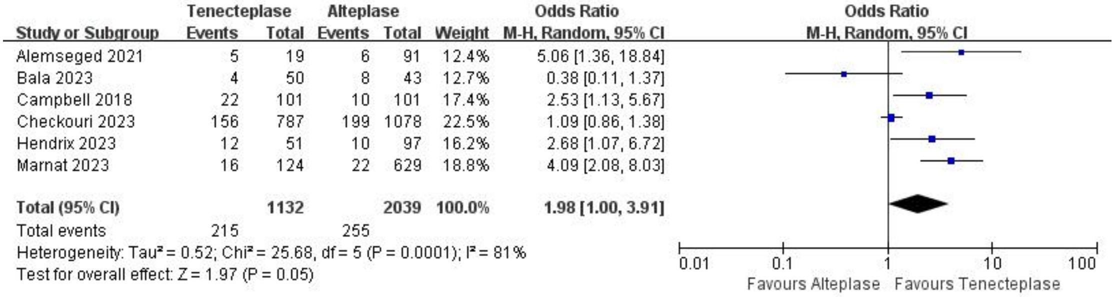

(B) sICH

Study or Subgroup	Tenecteplase Events	Tenecteplase Total	Alteplase Events	Alteplase Total	Weight	Odds Ratio M-H, Random, 95% CI
Alemseged 2021	0	19	1	91	3.0%	1.55 [0.06, 39.41]
Bala 2023	1	50	0	43	3.0%	2.64 [0.10, 66.41]
Campbell 2018	1	101	1	101	4.1%	1.00 [0.06, 16.21]
Hendrix 2023	2	51	1	97	5.3%	3.92 [0.35, 44.29]
Marnat 2023	14	124	69	629	84.6%	1.03 [0.56, 1.90]
**Total (95% CI)**		**345**		**961**	**100.0%**	**1.15 [0.66, 2.02]**
Total events	18		72			

Heterogeneity: Tau² = 0.00; Chi² = 1.40, df = 4 (P = 0.84); I² = 0%

Test for overall effect: Z = 0.50 (P = 0.62)

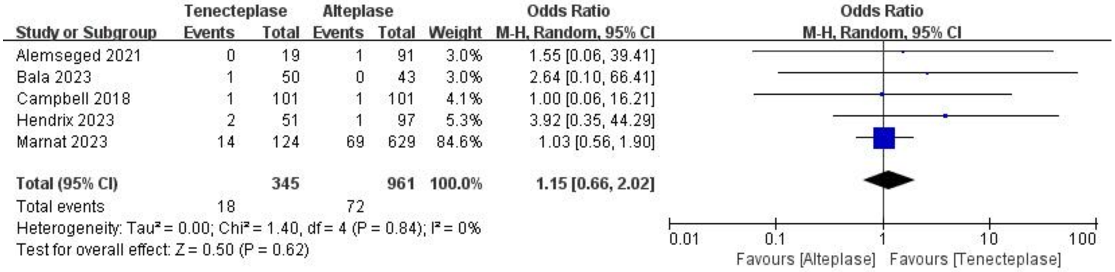

(C) ICH

Study or Subgroup	Tenecteplase Events	Tenecteplase Total	Alteplase Events	Alteplase Total	Weight	Odds Ratio M-H, Random, 95% CI
Alemseged 2021	0	19	3	91	4.7%	0.65 [0.03, 13.07]
Bala 2023	11	50	14	43	24.2%	0.58 [0.23, 1.47]
Campbell 2018	6	101	5	101	18.3%	1.21 [0.36, 4.11]
Hendrix 2023	3	51	7	97	15.6%	0.80 [0.20, 3.25]
Marnat 2023	82	124	295	629	37.2%	2.21 [1.48, 3.31]
**Total (95% CI)**		**345**		**961**	**100.0%**	**1.16 [0.58, 2.31]**
Total events	102		324			

Heterogeneity: Tau² = 0.29; Chi² = 8.49, df = 4 (P = 0.08); I² = 53%

Test for overall effect: Z = 0.41 (P = 0.68)

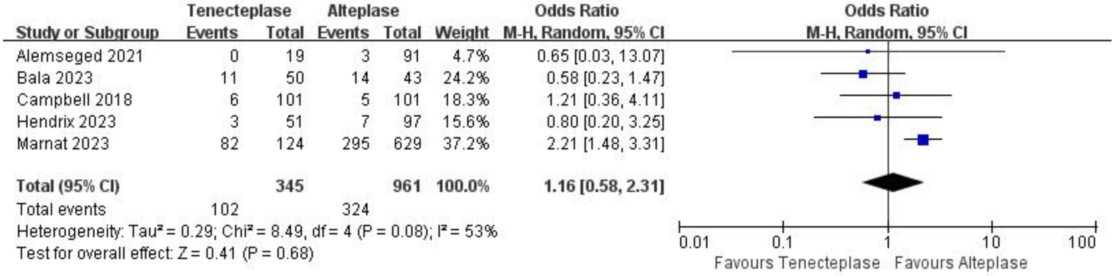

Supplementary File 4 Forest plots for primary outcomes including only peer reviewed articles

sICH: Symptomatic intracranial hemorrhage, ICH: Intracranial hemorrhage

(A) Functional Independence at 90 Days

Study or Subgroup	Tenecteplase Events	Tenecteplase Total	Alteplase Events	Alteplase Total	Weight	Odds Ratio M-H, Random, 95% CI
Alemseged 2021	9	19	43	91	7.0%	1.00 [0.37, 2.70]
Bala 2023	27	50	25	43	10.2%	0.85 [0.37, 1.92]
Campbell 2018	65	101	52	101	21.7%	1.70 [0.97, 2.99]
Hendrix 2023	28	51	58	97	14.7%	0.82 [0.41, 1.62]
Marnat 2023	61	124	296	629	46.4%	1.09 [0.74, 1.60]
**Total (95% CI)**		**345**		**961**	**100.0%**	**1.11 [0.86, 1.45]**
Total events	190		474			

Heterogeneity: Tau² = 0.00; Chi² = 3.43, df = 4 (P = 0.49); I² = 0%
Test for overall effect: Z = 0.81 (P = 0.42)

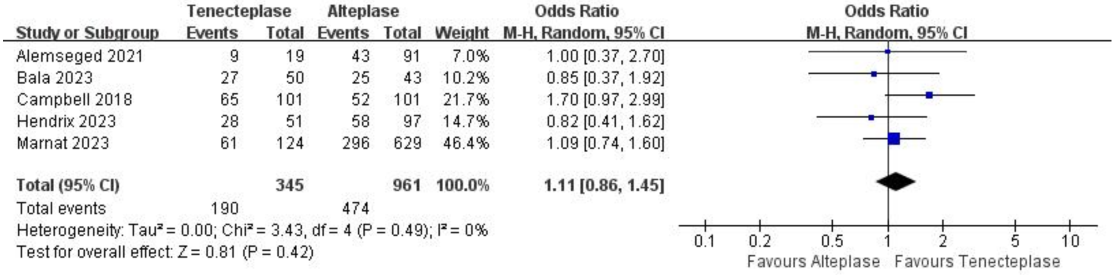

(B) Mortality within 90 Days

Study or Subgroup	Tenecteplase Events	Tenecteplase Total	Alteplase Events	Alteplase Total	Weight	Odds Ratio M-H, Random, 95% CI
Bala 2023	9	50	7	43	13.1%	1.13 [0.38, 3.34]
Campbell 2018	10	101	18	101	22.5%	0.51 [0.22, 1.16]
Hendrix 2023	9	51	20	97	20.3%	0.82 [0.34, 1.97]
Marnat 2023	14	124	114	629	44.1%	0.57 [0.32, 1.04]
**Total (95% CI)**		**326**		**870**	**100.0%**	**0.66 [0.44, 0.97]**
Total events	42		159			

Heterogeneity: Tau² = 0.00; Chi² = 1.79, df = 3 (P = 0.62); I² = 0%
Test for overall effect: Z = 2.09 (P = 0.04)

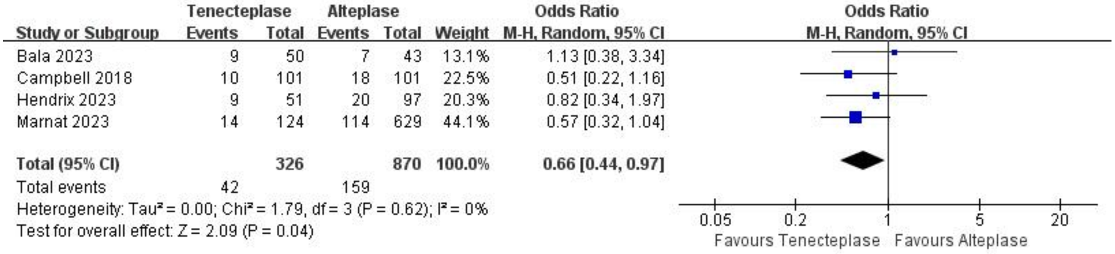

Supplementary File 5 Forest plots for secondary outcomes including only peer reviewed articles

(1)

Excluding Single Study	Early Recanalization		
	OR (95%CI)	I^2, %	P Value
Original	2.02 (1.20, 3.38)	73	0.008
Excluding Ainz Gomez 2021	2.19 (1.19, 4.02)	76	0.01
Excluding Alemseged 2021	1.85 (1.09, 3.13)	72	0.02
Excluding Bala 2023	2.35 (1.39, 3.96)	72	0.001
Excluding Campbell 2018	1.96 (1.10, 3.48)	74	0.02
Excluding Checkouri 2023	2.30 (1.35, 3.92)	55	0.002
Excluding Cruz Culebras 2021	1.96 (1.16, 3.31)	75	0.01
Excluding Hendrix 2023	1.95 (111, 3.43)	74	0.02
Excluding Marnat 2023	1.76 (1.07, 2.90)	63	0.03
Excluding Vetra 2022	1.91 (1.10, 3.32)	74	0.02

(2)

Excluding Single Study	ICH		
	OR (95%CI)	I^2, %	P Value
Original	1.21 (0.66, 2.25)	41	0.54
Excluding Campbell 2018	1.16 (0.53, 2.51)	52	0.71
Excluding Marín 2023	1.16 (0.58, 2.31)	53	0.68
Excluding Hendrix 2023	1.28 (0.63, 2.51)	46	0.49
Excluding Alemseged 2021	1.23 (0.63, 2.39)	51	0.55
Excluding Bala 2023	1.91 (1.33, 2.75)	0	0.0005
Excluding Marnat 2023	0.80 (0.43, 1.49)	0	0.48

Supplementary File 6 (1) Sensitivity analysis of early recanalization excluding single studies
(2) Sensitivity analysis of ICH excluding single studies.
